# Molecular phenotyping using networks, diffusion, and topology: soft tissue sarcoma

**DOI:** 10.1038/s41598-019-50300-2

**Published:** 2019-09-27

**Authors:** James C. Mathews, Maryam Pouryahya, Caroline Moosmüller, Yannis G. Kevrekidis, Joseph O. Deasy, Allen Tannenbaum

**Affiliations:** 1 Department of Medical Physics, Memorial Sloan-Kettering Cancer Center, New York, USA; 2 Department of Chemical and Biomolecular Engineering, Johns Hopkins University, Baltimore, USA; 3 Departments of Computer Science and Applied Mathematics & Statistics, Stony Brook University, Stony Brook, USA

**Keywords:** Sarcoma, Cellular signalling networks

## Abstract

Many biological datasets are high-dimensional yet manifest an underlying order. In this paper, we describe an unsupervised data analysis methodology that operates in the setting of a multivariate dataset and a network which expresses influence between the variables of the given set. The technique involves network geometry employing the Wasserstein distance, global spectral analysis in the form of diffusion maps, and topological data analysis using the Mapper algorithm. The prototypical application is to gene expression profiles obtained from RNA-Seq experiments on a collection of tissue samples, considering only genes whose protein products participate in a known pathway or network of interest. Employing the technique, we discern several coherent states or signatures displayed by the gene expression profiles of the sarcomas in the Cancer Genome Atlas along the TP53 (p53) signaling network. The signatures substantially recover the leiomyosarcoma, dedifferentiated liposarcoma (DDLPS), and synovial sarcoma histological subtype diagnoses, and they also include a new signature defined by activation and inactivation of about a dozen genes, including activation of serine endopeptidase inhibitor *SERPINE1* and inactivation of TP53-family tumor suppressor gene *TP73*.

## Introduction

Modern biological investigations often result in dense, high-dimensional datasets describing genes, proteins, mutations, or other variables. A near universal problem arises as the dimensionality of the data grows: how can the data be investigated in a relatively unbiased manner, to expose underlying clusters and relational structure? To date, there is a lack of robust techniques for exposing the structure of biological data in an unbiased, agnostic fashion. Biological relevance is maintained in this work by considering known pathways as an underlying guide. The technique we describe involves network geometry via the Wasserstein distance^1,2^, global spectral analysis in the form of diffusion maps^3^, and topological data analysis using the Mapper algorithm^4^. We apply the technique to gene expression profiles along gene sets participating in known pathways. We discern several coherent states or signatures displayed by the gene expression profiles of The Cancer Genome Atlas (TCGA) sarcoma project along the TP53 signaling network. The signatures substantially recover the leiomyosarcoma, dedifferentiated liposarcoma (DDLPS), and synovial sarcoma histological subtype diagnoses, and they also include a new signature defined by activation and inactivation of about a dozen genes, including activation of serine endopeptidase inhibitor *SERPINE1* and inactivation of TP53-family tumor suppressor gene *TP73*.

The mechanisms that intervene between DNA sequence genotype and overall cell phenotype are complex, including the presence of transcription factors, the chemistry of the cell microenvironment, and epigenetic factors like phosphorylation and methylation. We focus on the determination of transcriptomic molecular phenotypes. The simplest molecular phenotypes are defined by single marker genes, like the estrogen receptor (ER), progesterone receptor (PR), or human epidermal growth factor receptor 2 (HER2/ERBB2) status of breast carcinomas. In general a comparatively large number of genes must be considered simultaneously.

Methods falling under the heading of Genome Wide Association Studies (GWAS, typically concerning mutational profiles) or Gene Set Enrichment Analysis (GSEA, typically concerning gene expression profiles) take into account data concerning a large number of genes to ascertain statistical significance with respect to given known outcomes or endpoints such as disease states. In general, they do not attempt to discern coherent states in gene expression quantification profiles in an “unsupervised” manner. That is, these methods do not ascertain existing apparent molecular phenotypes, but rather impose or design molecular phenotypes specifically to serve as predictors for variables of ultimate interest like prognosis.

Seemann, Shulman, and Gunaratne^5^ employ degree 0 persistent homology towards the end of unsupervised analysis, leveraging the robustness of Topological Data Analysis (TDA) techniques for unsupervised clustering. One could also use various established unsupervised clustering algorithms such as hierarchical clustering or *k*-mean optimization methods, optionally preceded by dimensional reduction techniques like Principal Component Analysis (PCA), *t*-distributed Stochastic Neighbor Embedding (t-SNE), or Multi-Dimensional Scaling (MDS). Note, however, that hierarchical clustering has the drawback that the output of the algorithm strongly underdetermines the usual heat map visual representation. Every branch of the hierarchy tree creates an ambiguity in the order in which the samples are displayed.

From the topological point of view, however, any method within the clustering paradigm is order 0 in the sense that it summarizes a dataset in terms of a finite/discrete set of disjoint categories, a “space” of dimension 0. Lockwood and Krishnamoorthy^6^ advocate “higher order” methods, e.g. degree 1 persistent homology, extracting 1-dimensional features in the space charted by the data points, roughly in order to take account of the relations between categories and not just the categories themselves. One major difficulty with this approach is that homology classes are defined by *cycles*; topological features which are not cycles, such as *relative cycles* or branches, are not detectable with existing tools (see^7^ for general background on topology, or^8^ for background on TDA methods). A second major difficulty is that homology classes do not have canonical representative cycles. This means that in theory an almost arbitrary subset of the points of a point cloud can appear along the path of a cycle belonging to an observed persistent 1-homology class. In other words, while persistent homologies are certainly evidence of important dataset-specific global features, there is an unsolved problem of interpretability of such features.

Camara, Emmett, and Rabadan^9^ calculate persistent 1-homologies in evolutionary/phylogenetic data, surmounting both of these difficulties simultaneously by interpreting the presence of non-trivial cycles (closed loops), and not the internal structure of their representative cycles *per se*, as an indication of the presence of genetic recombination events.

We largely follow Nicolau *et al*.^4^ in that we use the Mapper algorithm to map our point clouds onto summary spaces of dimension 1, graphs or networks. This algorithm can be regarded as a discrete version of the Morse-theoretic analysis of a smooth manifold with respect to a height function (called the filter function). Nicolau *et al*. heavily de-sparsify the point clouds, in order to avoid the normal preprocessing step of dimensional reduction (virtually always required for biological datasets), and employ a carefully designed deviation-from-normal filter function in accordance with what they call the Progression Analysis of Disease paradigm. We take a slightly different tack: First, we perform a biologically-motivated intermediate-scale dimensional reduction by considering only those genes participating in well-known pathways (we use the Kyoto Encyclopedia of Genes and Genomes). Next, we replace the ordinary Euclidean distance metric between gene expression profiles with alternative metrics, especially a version of the Wasserstein 1-metric which takes account of curated knowledge of the network structure linking the genes (coordinates). Then we employ the dimensional reduction and analysis technique of diffusion maps^3^ to regularize the point cloud with respect to intrinsic or characteristic global geometry. We have found that this process results in datasets with favorable properties for the application of Mapper and the interpretation of its resultant graph summaries.

## Methods

We take as our primary input a gene expression quantification sample set, as a point cloud $S\subset {{\mathbb{R}}}^{N}$, and an influence, regulation, or pathway network *G* relating the *N* genes which label the coordinates. Optionally, we include an additional control dataset $C\subset {{\mathbb{R}}}^{N}$, or a function $f:S\to {\mathbb{R}}$ with the interpretation as an experimentally-determined “degree of progression” with respect to some process (e.g., a disease process).

The output is a list of coherent states or molecular phenotypes, characterized by activated, inactivated, and equivocally-activated genes. We now enumerate the steps of our pipeline. Details will be given in subsequent sections.

1. **Normalize the values of** ***S***
2. **Restrict/project** ***S*** **to the genes appearing in the gene network** ***G***
3. **Calculate a network-based distance metric between samples**
4. **Evaluate a diffusion map**
5. **Perform the Mapper algorithm on the re-mapped** ${\boldsymbol{S}}\subset {{\mathbb{R}}}^{{\boldsymbol{M}}}$
6. **Extract and process the state graph**
7. **Plot heat maps and discern coherent states**

### Normalize the values of *S*

We must ensure that the values of *S* represent gene expression quantification, for example, FPKM (Fragments Per Kilobase Million) or TPM (Transcripts Per Kilobase) values as a result of a high-throughput sequencing pipeline. These values correspond roughly to the concentration of RNA transcripts in the tissue samples, typically across many cells for each sample (bulk sequencing) though sometimes for single cells.

Optionally, for comparison between genes and for the purposes of image-rendering, for each of the *N* genes, we replace the values of *S* in the corresponding coordinate by a truncated translated *z*-score, $x\mapsto (x-\mu )/(3\sigma )+0.5$, where *μ* and *σ* are the mean and standard deviation of the values for this coordinate and *x* is a typical value of this coordinate. The resulting values will be substantially normalized to lie on a scale from 0 to 1 with mean 0.5.

### Restrict/project *S* to the genes appearing in the gene network *G*

We select a network *G* whose nodes correspond to genes whose presence or absence constitutes participation in a coordinated function or process of interest, and whose edges represent the coordinating relationships. Omit the expression values for genes not participating in *G*. The networks we consider are the KEGG (Kyoto Encyclopedia of Genes and Genomes) pathways concerning cell cycle regulation, senescence, proliferation, apoptosis, and TP53 signaling.

### Calculate a network-based distance metric between samples

**Figure 1:**
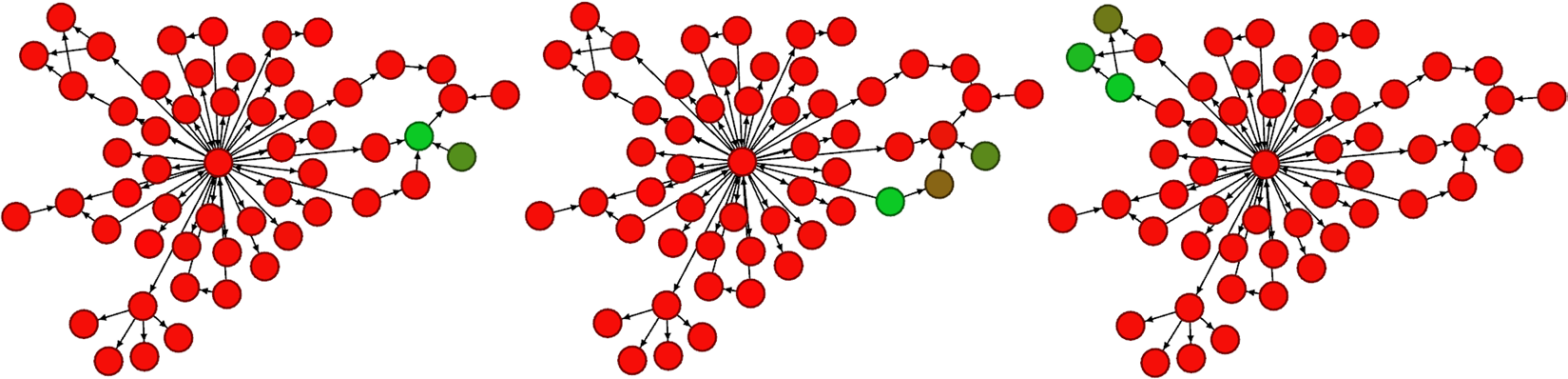
Left to right, distributions *s*_1_, *s*_2_, *s*_3_, for illustration. Red represents values close to zero, and green more positive. The Wasserstein 1-distance *d*(*s*_1_, *s*_2_) = 1.10 is much less than *d*(*s*_2_, *s*_3_) = 3.08, while the corresponding Euclidean distances are approximately equal to each other.

For each sample *s* ∈ *S*, define a probability distribution *p*_*s*_ on the set of nodes of *G* by interpreting the values of *s* (divided by their sum) as a probability density function. Alternatively, use a distribution on the nodes which is the invariant measure for a Markov chain stochastic process inferred from the values of the sample *s*, in the manner of^10^. It can happen that *G* is disconnected into several components, with no edges/links between components, in which case one should define separate distributions for each component. Note that this disconnection may be either genuine or an indication of missing biological knowledge, so that a network-connection inference method may be useful. For each component *c*, calculate the Wasserstein 1-metric or Earth Mover’s Distance *d*_*c*_(*s*, *s*′) between each pair *p*_*s*_ and *p*_*s*′_ with respect to the path-length metric on *c* (weighted by the reciprocal of strength-related edge weights, if present). Intuitively, the Earth Mover’s Distance measures the total effort needed, in the best case scenario, to displace one distribution into another, taking account of the ground point-to-point distance. This distance is illustrated for networks in Fig. 1. Technically, the Earth Mover’s Distance between two mass distributions on a common metric space is defined as the infimum of the total mass-weighted displacement among displacement functions from the space to itself which map the first mass distribution onto the second. Classically it is only defined if the total masses of both distributions are equal^1^. We use the “direct sum” formula to amalgamate these distances across components *c* into a single distance for each pair of samples (*s*, *s*′):

$$d(s,s^{\prime} )=\sqrt{\sum _{c}{d}_{c}{(s,s^{\prime} )}^{2}}.$$

The Wasserstein 1-metric employed in this way is perhaps the simplest alternative to the standard Euclidean metric for which a network or pathway structure relating the coordinates is in some way taken into account. The principal benefit of this metric is that it greatly increases the distance between two samples in comparison with the Euclidean metric in case the main activity of one sample takes places in an area of the network very far from the area of main activity of the other sample. One conceivable disadvantage is that isolated changes to a given sample, say in the expression of a single gene, can have an outsized effect on the Wasserstein 1-distance of the displacement. We also caution that since the KEGG database networks are enriched with nodes for various compounds and macromolecules in addition to protein gene products, an analysis which considers only gene expressions will not take advantage of the full KEGG pathways and may have some misleading consequences. To compute the Wasserstein distance, we used the Hungarian algorithm^2^.

### Evaluate a diffusion map

In order to reduce the dimension and complexity of the dataset *S*, while preserving key information for subsequent analysis, we apply diffusion maps^3,11^. This manifold learning technique provides a global parametrization of a low-dimensional, possibly nonlinear manifold on which the high-dimensional data is assumed to lie. Such an embedding is obtained by spectral properties (eigenvalues and eigenvectors) of the graph Laplacian on a certain weighted graph with nodes *S*. Its eigenvectors can be used as a coordinate system on the dataset *S*, which is justified by the fact that they approximate the eigenvectors of the Laplace-Beltrami operator of the underlying manifold^3,12^. See also some recent work^13^ for employing diffusion map techniques for data whose “points” are weighted graphs.

Following^11^, for data samples *s*_*i*_, *s*_*j*_ ∈ *S* we define a connectivity matrix *W* using a Gaussian kernel:

1 $${W}_{ij}=\exp (-\frac{{d}^{2}({s}_{i},{s}_{j})}{\varepsilon }),$$

where *d* is the Wasserstein 1-metric as defined in^1^ and *ε* is the kernel scale parameter. The kernel is intended to capture the features of the underlying dataset and it is therefore reasonable to choose the metric *d* and the scale parameter *ε* based on the application. The parameter *ε* defines a local connectivity scale, in the following sense: If *s*_*j*_ is in the *ε*-ball around *s*_*i*_, the kernel induces high weight between *s*_*i*_ and *s*_*j*_. Otherwise the weights are negligible. We can choose *ε* to be almost any value between the minimum and maximum among the pairwise squared distances (*d*(*s*_*i*_, *s*_*j*_))^2^.

Define an adapted kernel

2 $$\tilde{W}={D}^{-1}W{D}^{-1}$$

with *D* the diagonal matrix ${D}_{ii}={\sum }_{j=1}^{N}{W}_{ij}$, *N* = #*S*. We use the adapted kernel (2) instead of (1), corresponding to the choice *α* = 1 in the family of kernel normalizations presented in^3,14^, to recover the Riemannian geometry of the underlying data independently of the data sampling.

We build a weighted graph with node set *S* and weights ${\tilde{W}}_{ij}$ of the edge connecting *s*_*i*_ to *s*_*j*_. Now apply weighted graph Laplacian normalization to $\tilde{W}$

3 $${\tilde{D}}^{-1}\tilde{W},$$

with $\tilde{D}$ the diagonal matrix ${\tilde{D}}_{ii}={\sum }_{j=1}^{N}{\tilde{W}}_{ij}$. The associated graph Laplacian is given by

4 $$L=I-{\tilde{D}}^{-1}\tilde{W}.$$

**Figure 2:**
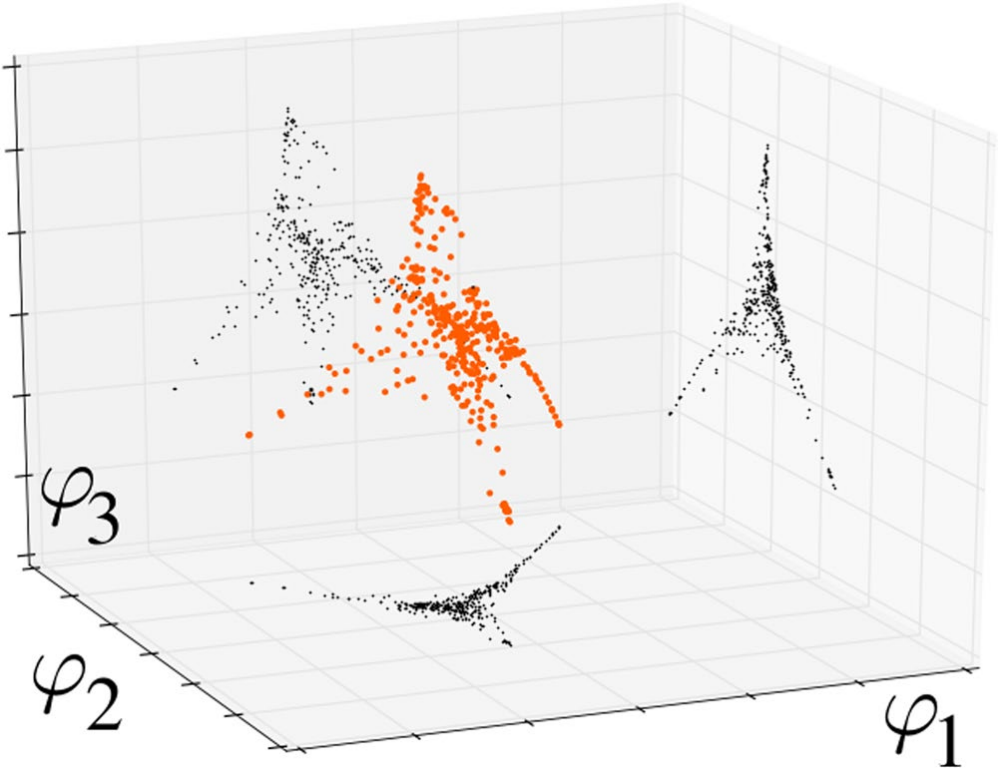
The diffusion re-mapped images of the gene expressions of 355 adipose visceral omentum tissue samples from the GTEx database. The first three eigenfunctions of the diffusion operator are used, to make a three-dimensional plot.

We compute the eigenvalues 1 = *λ*_1_ ≥ |*λ*_2_| ≥ … ≥ |*λ*_*N*_| and eigenvectors *φ*_1_, …, *φ*_*N*_ of ${\tilde{D}}^{-1}\tilde{W}$. These eigenvectors provide an embedding of the data into a space of dimension *M* < *N* (for example, as shown in Fig. 2):

5 $$\Phi ({s}_{i})=[{\phi }_{1}(i),\ldots ,{\phi }_{M}(i)].$$

We reiterate that rather than using the Euclidean distance between samples *s*_*i*_ and *s*_*j*_, we select the “more informed” 1-Wasserstein network metric. Unlike dimensional reduction techniques like Principal Component Analysis or Local Linear Embedding, but in common with *t*-SNE or MDS, the technique of diffusion maps can function on arbitrary intrinsic-metric representations of the data of the point cloud and does not require this point cloud to be presented in some Euclidean space. We prefer diffusion maps over t-SNE or MDS because as far as we know the latter are not guaranteed to recover the intrinsic manifold degrees of freedom of the original dataset, while diffusion maps are so guaranteed in principle. In practice biological datasets of present interest seem to represent processes of sufficient complexity that precise quantitative accounting for the relationships between all of the variables is rarely proposed, so such theoretical considerations are arguably premature.

We remark that dimensional reduction of gene expression data via diffusion maps is also suggested e.g. in^15^, where the authors combine diffusion maps with a neural network clustering method to differentiate between different types of small round blue-cell tumors.

### Perform the Mapper algorithm on the re-mapped ${\boldsymbol{S}}\subset {{\mathbb{R}}}^{{\boldsymbol{M}}}$

This algorithm results in a simplicial complex, in some sense modeling the mesoscopic-scale topology of the support space for the collection of samples or states *S*. It works by (1) dividing the point cloud into overlapping “slices” by binning the values of a chosen filter function $f:S\to {\mathbb{R}}$ into overlapping bins, (2) clustering the points of each slice (e.g. with single-linkage clustering), and (3) linking pairs (or tuples) of clusters by edges (or higher-dimensional simplices) depending on the amount of overlap between clusters.

A reasonable choice for the filter function is a deviation function devised in comparison with a control dataset *C*, roughly as in Nicolau *et al*.^4^, e.g. the Mahalanobis distance function adapted to *C* in case the size of *C* is sufficiently large in comparison to the dimension *M*. We often use the general-purpose network centrality measure available in Daniel Müllner’s Python Mapper implementation (http://danifold.net/mapper/).

The algorithm requires the choice of certain resolution or scale parameters: the number *n*_*f*_ of filter-level-set bins and a threshold *t* for the single-linkage clustering algorithm applied to each slice. One should select these parameter values intermediate between the extreme values which completely divide the sample set into isolated clusters and those which completely merge the sample set into a single cluster. In practice a narrow range of such values exists.

Applying the Mapper algorithm in this way is an *ad hoc* (case by case) form of ascertaining persistent topological features. *Persistence* is meant in roughly the same sense as the technique of *persistent homology*. Though persistent homology would ordinarily determine preferred values for parameters like *n*_*f*_, existing persistent homology tools are seemingly inapplicable to our setting. This is because it is multi-dimensional in that the simplicial complexes of interest depend on multiple parameters *n*_*f*_ and *t*, and because a well-defined relation of containment or mapping between the complexes across parameter values is not apparent. Nevertheless appropriate values for *n*_*f*_ and *t* are normally apparent.

### Extract and process the state graph

**Figure 3:**
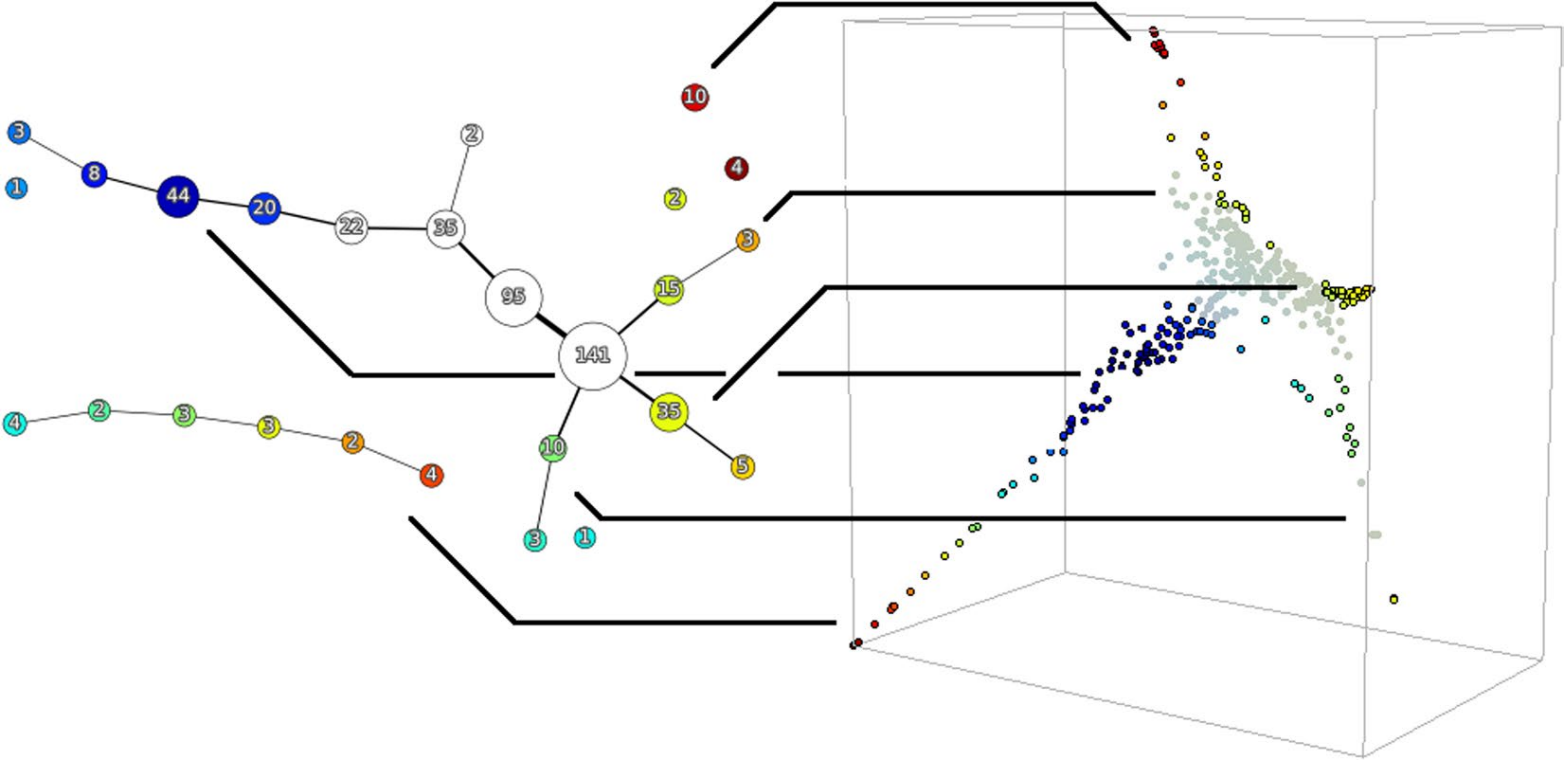
The Mapper state graph of the diffusion re-mapped 355-sample GTEx dataset of Fig. 2, with branches highlighted. For illustration, the “core” is not highlighted. The numerical labels indicate the number of samples in a cluster. The color indicates the value of the filter function which was used to seed the Mapper algorithm (a nearest-neighbor network closeness centrality in this case).

Next, we consider the graph which is the 1-skeleton of the simplicial complex resulting from the Mapper algorithm. We decompose it into linear paths, and concatenate these paths for display. An example state graph is shown in Fig. 3.

### Plot heat maps and discern coherent states

We order the samples within each node of the state graph according to the filter values. This ordering is combined with the concatenated linear path structure for an ordering of the samples *S* along one or both axes of a two-dimensional plot of:

- the expression values
- the correlations with subpopulations defined by discrete covariates
- 1-Wasserstein distance matrix
- diffusion map Euclidean distance matrix

Salient coherent states may appear in the expression heat map defined by patterns of activation and inactivation of particular genes, especially near the two extreme values for the filter function. This may require approximate dichotomization of the expression values (i.e. increasing the contrast, in the terminology of image processing).

From the point of view of topological data analysis, the most interesting states are ones which are not separated by the chosen filter function alone, but are nevertheless distinguished by branching of the state graph. We caution that although the topological aspect of this pipeline has the benefit of insensitivity to dimensionality, functioning well even in very high-dimensional settings, it sometimes provides little insight beyond that already provided by the spectral analysis or diffusion map when the number of samples is small. On the other hand, Mapper seems to have the potential to function well even without diffusion maps preprocessing or gene network analysis, provided that a filter function can be chosen that brings sufficiently rich outside information into the analysis.

## Results

**Figure 4:**
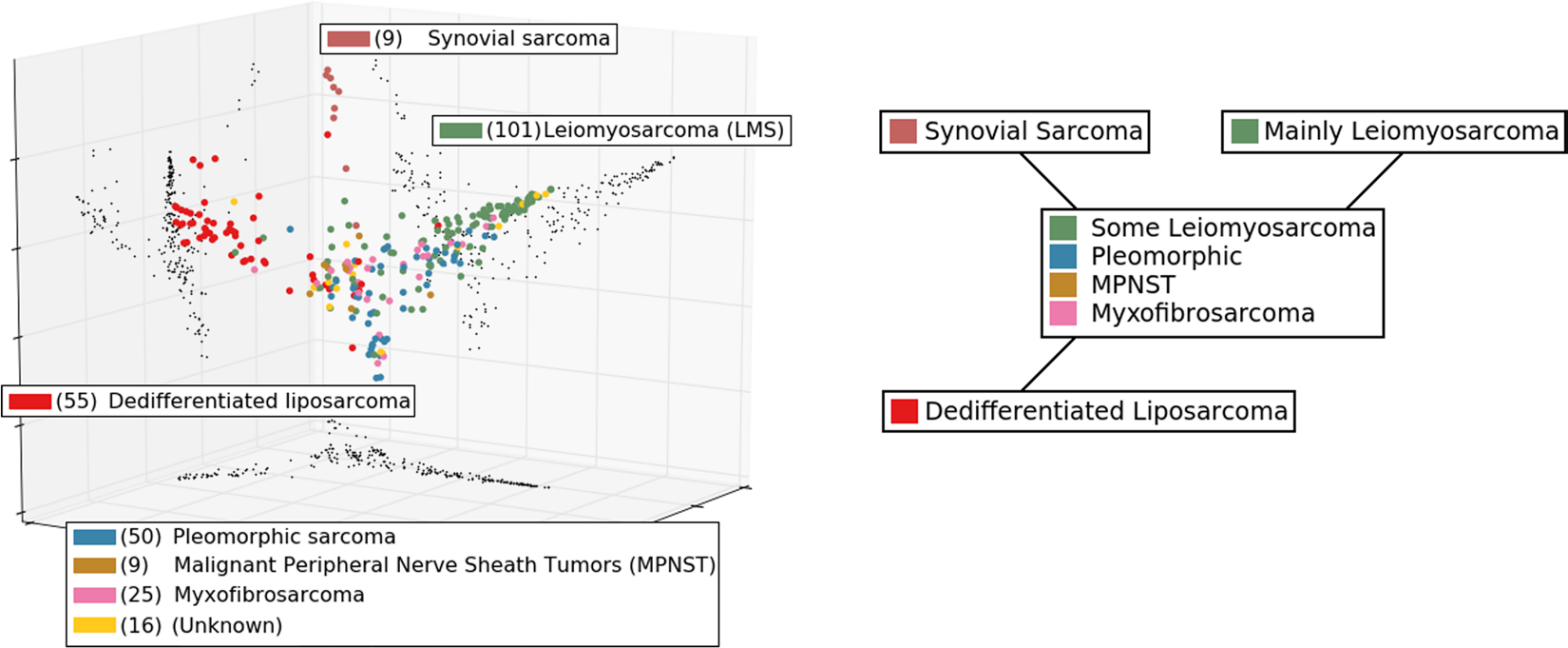
(Left) The diffusion re-mapped images of the 265 gene expression profiles from the TCGA sarcoma project, restricted to the TP53 signaling network defined in the KEGG database^24,25^. The first, second, and fourth eigenvectors were used. (Right) A schematic of the state graph summary produced by the Mapper algorithm.

**Figure 5:**
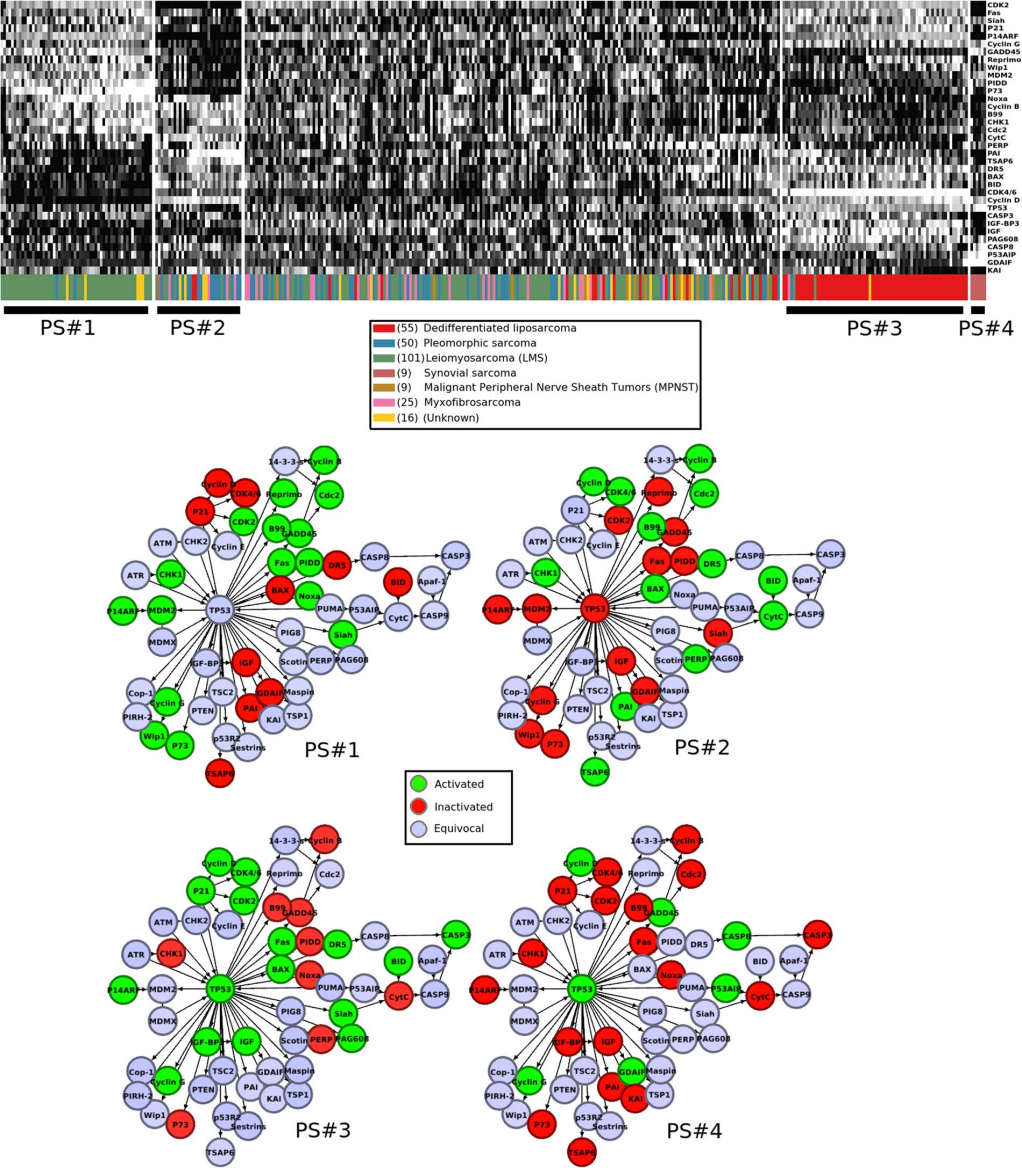
Four coherent states of the KEGG TP53 signaling network displayed by subsets of the TCGA sarcoma samples, shown superimposed on the network. The gene names are shown as they appear in the KEGG network.

The application of the network-metric/diffusion-map/Mapper pipeline to the 265 gene expression profiles of the samples of the TCGA sarcoma project demonstrates the basic efficacy of the method. The state graph is shown in Fig. 4, and the heatmaps are shown in Fig. 5, with tissue classification.

### Discussion of sarcoma states

We refer to the KEGG TP53 signaling pathway and indicate in parentheses the gene names appearing there, following official HUGO gene symbols. Italics indicate protein products.

The state PS#1 (TP53/P53 Signaling 1) consists almost entirely of samples tagged for leiomyosarcoma, meaning that tissue pathology determined a derivation from smooth muscle cells. As expected, high levels of CCNG1 (cyclin G), PPM1D (Wip1), and TP73 (P73), as well as *MDM2* are all negatively regulating *TP53*, which is not substantially activated. Arrest of the G1 and G2 phases should be frequently triggered since *CDK2*, *CCNB1* (cyclin B), and *CDK1* (CDC2), are all activated. Although *FAS*, *PIDD1* (PIDD), *PMAIP1* (NOXA), and *SIAH1* (SIAH) are substantially activated, the apoptosis pathway for which they are precursors is not, including low levels of *BAX*, the death receptor protein *TNFRSF10B* (DR5), *BID*, *CYCS* (cytochrome c), and all caspases. Apoptosis seems to have been largely evaded.

PS#3 consists almost entirely of samples tagged for dedifferentiated liposarcoma (DDLPS), and conversely almost all of the dedifferentiated liposarcomas among the 265 samples display state PS#3. We emphasize for clarity that all of the states were determined in an entirely unsupervised manner, with no input from the histological classification. *TP53* is strongly activated, and its negative regulator *MDM2* seems to be repressed by *CDKN2A* (P14ARF). A large number of the elements of the normal apoptosis signaling pathway are activated: *FAS*, *BAX*, *TNFRSF10B*, *BID*, *ZMAT3* (PAG608), and *SIAH1*. The downstream caspase *CASP3* is substantially activated. Apoptosis may occur in dedifferentiated liposarcomas at comparatively high rates. Alternatively, see^16^ for a discussion of situations where normally apoptotic caspases are non-lethal to the cell. Substantial activation of *CDKN1A* (P21) is not inhibiting *CDK4*/6 or *CDK2* as expected; rather *CDK4*/6 over-expression is the most salient characteristic of state PS#3. According to Binh *et al*.^17^, over-expression of *CDK4 and MDM2* is known to be a reliable diagnostic marker for well-differentiated liposarcoma (not represented in the TCGA sarcoma project).

Note that both leiomyosarcoma and DDLPS subtypes are known to exhibit complex karyotypes, with no apparent characteristic mutation. This seems to be part of the reason why they were selected for inclusion in the TCGA sarcoma project (https://cancergenome.nih.gov/cancersselected/Sarcoma). Nevertheless the coherent states PS#1 and PS#3 show that the expression profiles for these subtypes are more organized than their mutational profiles. We remark that ordinary unsupervised hierarchical clustering substantially reproduces these results, with somewhat less coherence among the apparent states.

On the other hand, synovial sarcoma is known to be well-characterized by a specific translocation resulting in gene fusion of *SYT* with either *SSX1*, *SSX2*, or *SSX4*^18^. So it is not surprising that there is a coherent state, PS#4, displayed by precisely the synovial sarcomas.

Finally, we consider the state PS#2. It does not consist mainly of any one histopathological subtype. The most obvious feature is that *TP53* and almost all of its normal positive regulation targets are inactivated, despite high levels of *CHEK1* (CHK1) potentially indicating DNA damage. Nearly all of the markers for *TP53* negative feedback regulation are strongly inactivated, including *CCNG1*, *PPM1D*, *TP73*, and *MDM2*. *CDKN1A* inactivation is consistent with the appearance of *CCND1* (cyclin D) and *CDK4*/6. With respect to the upstream elements of the apoptosis signaling pathway, we observe in state PS#2 the opposite behavior from the state PS#1, namely that *FAS*, *PIDD1*, *SIAH1*, and possibly *PMAIP1* are absent, but *BAX*, *PERP*, *TNFRSF10B*, and *BID* transcripts are all present.

A cBioPortal^19^ query of the TCGA sarcomas displaying the *TP73*-/*SERPINE1*+ expression pattern of PS#2 reveals high probability of loss of some portion of chromosome 18q. *SERPINB3*, which is located on 18q, and *TP73* are both associated with negative regulation of JUN kinase activity (GO:0043508) according to the human Gene Ontology Annotation database^20^.

This suggests the following narrative to explain the molecular mechanisms driving the cancers in state PS#2. Damage to *SERPINB3* on chromosome 18q disrupts serine/cysteine-type endopeptidase inhibitor activity, which is then restored by *SERPINE1* upregulation by some intermediate process. Normal SERPINB3 would in addition inhibit JUN kinase activity, but this inhibitory function is *not* restored by *SERPINE1* upregulation. TP73 also normally inhibits JUN kinase activity. Unchecked JUN kinase activity may then be the main driver of tumor cell proliferation and transformation^21^ in state PS#2 since effective *TP73* and *SERPINB3* both seem to be absent.

The high degree of *CCND1* activity of PS#2 is consistent with this hypothesis, since JUN induces transcription of *CCND1*^22^.

Note that a proto-oncogenic role for JUN has long been suspected, and its actual function is complex, including alternately pro- and anti-tumor behaviors depending on context^23^. In this specific case, our finding answers the call of Messoussi *et al*.^23^ to delineate patients that would potentially benefit from JNK (c-Jun N-terminal kinase) inhibitors. In approximately 10% of soft-tissue sarcomas, largely irrespective of histological subtype and possibly independent of JUN amplification status, JNK inhibitors that can replace the inhibitory function no longer provided by *SERPINB3* may restore JNK activity to normal condition.

### Conclusion and future research directions

The network-metric/diffusion-map/Mapper pipeline uncovered some latent features of high-dimensional genomic sarcoma data in a relatively robust way. One promising direction for future research is the inference of phylogenetic trees via mutational data like Single Nucleotide Polymorphism (SNP) calls or gene amplifications and deletions in the evolutionary context. This context could be spatially dense single-tumor samples or single-patient metastasis or micrometastasis samples. Mapper would be especially adapted to the elucidation of branching/inheritance structures when the filter function is a suitable quantification of the deviation of a sample from a founder population. For example, a Hamming-type distance in the case of SNP sequences, which could also be used for the intrinsic metric between SNP sequences. As an alternative to the naive Hamming distance, a network-enriched distance could be obtained by means of linkage disequilibrium calculations.
